# Classification Performance of General-Purpose Multimodal Large Language Models Across Orthodontic Radiographic Tasks: A Comparative Study of ChatGPT, Gemini, and Claude

**DOI:** 10.3390/medicina62091714

**Published:** 2026-09-06

**Authors:** Nuri Can Tanrısever, Kutluhan Yılmaz, Ayşegül Dilara Güvenç Tokur, Merve Berika Kadıoğlu, Mahmut Kadıoğlu

**Affiliations:** 1 Department of Orthodontics, School of Dentistry, Ankara University, 06560 Ankara, Türkiye; 2 Independent Researcher, 06560 Ankara, Türkiye

**Keywords:** age determination by skeleton, artificial intelligence, cephalometry, large language models, orthodontics, radiography, panoramic

## Abstract

*Background and Objectives*: General-purpose multimodal large language models (MLLMs) can interpret radiographic images, but their classification performance across orthodontic tasks remains uncertain. This study compared the classification performance of ChatGPT, Gemini, and Claude on lateral cephalometric, hand–wrist, and panoramic radiographs. *Materials and Methods*: This retrospective diagnostic accuracy study included 250 individuals, each contributing one lateral cephalometric, hand–wrist, and panoramic pretreatment radiograph (750 total). Reference classifications were established by two experienced orthodontists, with disagreements resolved by consensus. Lateral cephalometric radiographs were classified as skeletal Class I, II, or III based on the ANB angle according to Steiner analysis; hand–wrist radiographs as prepubertal, pubertal, or postpubertal; and panoramic radiographs as early mixed, late mixed, or permanent dentition. Each image was evaluated once by each AI platform using identical Turkish prompts in separate chat sessions. Classification accuracy, balanced accuracy, macro-F1, class-specific metrics, and reference agreement were assessed. Generalized estimating equations (GEE) assessed platform, radiograph type, and interaction effects on correct classification. *Results*: ChatGPT had the highest hand–wrist accuracy (81.6%; 95% CI, 76.3–85.9), whereas Gemini had the highest panoramic accuracy (92.8%; 95% CI, 88.9–95.4). Lateral cephalometric accuracies were 70.0%, 64.4%, and 64.8% for ChatGPT, Gemini, and Claude, respectively, with no significant interplatform difference (*p* = 0.336). The platform × radiograph type interaction was significant (Wald χ^2^ = 42.52; df = 4; *p* < 0.001). Agreement with the reference standard was highest for ChatGPT on hand–wrist radiographs (κw = 0.758) and Gemini on panoramic radiographs (κw = 0.883). *Conclusions*: Classification performance was task- and platform-dependent, with no model consistently achieving the highest performance. For the predefined classification tasks, these models should not be used as standalone tools for these classification tasks. Their potential as decision-support tools requires prospective evaluation of AI-assisted clinician performance and external validation.

## 1. Introduction

Orthodontic diagnosis and treatment planning require the comprehensive evaluation of radiographic records in conjunction with clinical examination [1]. Lateral cephalometric radiographs are routinely used to assess sagittal and vertical skeletal relationships, hand–wrist radiographs to determine growth and developmental status, and panoramic radiographs to evaluate dental development, eruption patterns, and dentition stage. Information derived from these radiographs plays a critical role in characterizing malocclusion, determining appropriate treatment timing, and selecting the most suitable treatment approach [2,3,4,5].

Accurate and consistent interpretation of orthodontic radiographs requires clinical expertise and may be subject to interobserver variability. Consequently, increasing attention has been directed toward artificial intelligence (AI) applications aimed at reducing diagnostic variability, standardizing assessment, and supporting clinical workflows. Deep learning-based systems have been widely investigated for orthodontic applications, including cephalometric landmark detection, automated cephalometric analysis, skeletal maturation assessment, image segmentation, and orthodontic treatment planning. However, much of the available evidence is based on task-specific AI models developed for narrowly defined clinical purposes [6,7].

The emergence of general-purpose multimodal large language models (MLLMs), such as ChatGPT, Gemini, and Claude, has broadened the potential applications of AI in dental image interpretation. Unlike task-specific AI systems, these models can analyze images together with natural-language prompts without additional task-specific training and can be applied to a variety of clinical tasks, which may broaden their potential applicability to orthodontic image interpretation and related decision-support contexts. However, the extent to which performance in isolated image-based tasks translates into clinically meaningful decision support remains to be established [8].

Large language models were initially investigated in orthodontics primarily for text-based applications, such as answering clinical questions and generating treatment plans [9,10]. With the development of multimodal capabilities, general-purpose MLLMs have also been evaluated in image-based tasks, including skeletal maturation assessment, interpretation of lateral cephalometric images, and detection of radiographic findings on panoramic radiographs [11,12,13,14]. Current evidence suggests that their diagnostic performance and agreement with expert assessments may vary according to the model and the specific image-based task being evaluated.

The diagnostic demands of these radiographic assessments differ substantially. Hand–wrist radiographs require recognition of morphological maturation indicators, panoramic radiographs require assessment of dental eruption and dentition patterns, whereas lateral cephalometric radiographs require interpretation of spatial relationships among craniofacial structures. Accordingly, performance observed in one radiographic task may not necessarily be generalizable to another.

An additional consideration is that general-purpose MLLMs are continuously evolving systems whose capabilities may change across model versions and over time. Therefore, head-to-head comparisons should be interpreted as assessments of the specific model versions available during the study period rather than as fixed characteristics of the platforms themselves.

Although recent studies have begun to directly compare general-purpose MLLMs [12,14], most available evidence remains focused on a single radiograph type or a specific diagnostic task. Evidence directly comparing ChatGPT, Gemini, and Claude across lateral cephalometric, hand–wrist, and panoramic radiographs within the same sample and under a standardized evaluation protocol remains limited. A head-to-head comparison using identical image-based prompts and a common expert-derived reference standard may provide a more controlled assessment of whether the relative classification performance of these models is dependent on the radiographic task.

Therefore, this study aimed to evaluate and compare the classification performance of ChatGPT, Gemini, and Claude on lateral cephalometric, hand–wrist, and panoramic radiographs. The null hypothesis was that classification performance would not differ according to AI platform or radiograph type and that there would be no platform × radiograph type interaction.

## 2. Materials and Methods

### 2.1. Study Design and Sample Selection

This retrospective diagnostic accuracy study was conducted using pretreatment lateral cephalometric, hand–wrist, and panoramic radiographs retrieved from the archives of the Department of Orthodontics, Faculty of Dentistry, Ankara University. Radiographic classifications generated by ChatGPT, Gemini, and Claude were compared with an expert-derived reference standard. The study protocol was approved by the Ankara University Ethics Committee for Non-Interventional Scientific Research (Meeting No. 20; 30 March 2026), and the study was conducted in accordance with the Declaration of Helsinki.

A total of 276 individuals with pretreatment records obtained between September 2021 and March 2024 were retrospectively screened. The screened records represented consecutive pretreatment cases within the defined study period, with no additional sampling procedure applied. Individuals were eligible if complete lateral cephalometric, hand–wrist, and panoramic radiographs belonging to the same pretreatment diagnostic record set were available and image quality was sufficient for diagnostic assessment. The same eligibility criteria were applied to all individuals irrespective of age or developmental stage, and no stage-specific sampling or age-based restriction was used. Radiographs were considered diagnostically acceptable when the anatomical structures required for the corresponding classification task were fully visualized, with sufficient sharpness and contrast and without substantial motion, positioning, or other artifacts that could interfere with interpretation. Twenty-six individuals were excluded because of missing radiographic records (*n* = 11), inadequate image quality (*n* = 10), craniofacial syndrome or congenital anomaly (*n* = 1), or a history of orthognathic surgery (*n* = 4). Following eligibility assessment, 250 individuals with a total of 750 radiographs were included in the final analysis.

### 2.2. Radiographic Acquisition and Image Preparation

**Figure 1:**
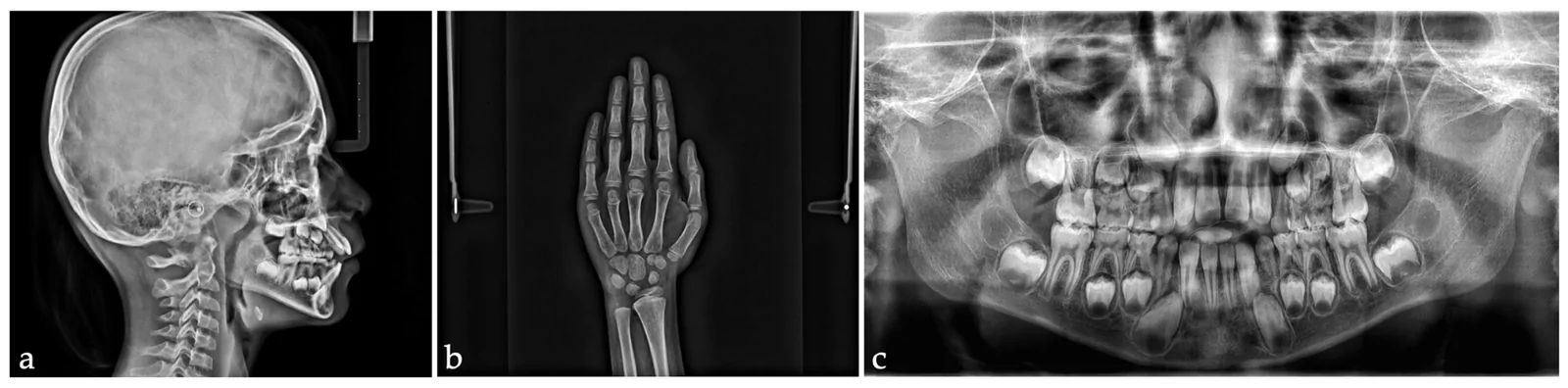
Pretreatment radiographic records from the same individual included in the study, illustrating the three radiographic tasks evaluated: (**a**) lateral cephalometric radiograph; (**b**) hand–wrist radiograph; and (**c**) panoramic radiograph.

All lateral cephalometric, hand–wrist, and panoramic radiographs (Figure 1) were acquired for routine orthodontic diagnostic purposes using the same digital imaging system (Carestream Dental 8100, Carestream Health Inc., Rochester, NY, USA) under the institution’s standard acquisition protocol. The available exposure parameters were 80 kVp, 10 mA, and an exposure time of 10 s. Additional modality-specific acquisition metadata, including image resolution and device-specific settings, were not consistently available in the archived records. Before evaluation, all images were anonymized, and personal identifiers were removed. The radiographs were originally acquired and archived in DICOM format and were exported as JPEG files from the institutional imaging system for AI evaluation. No investigator-applied resizing, cropping, or image enhancement was performed after export. Export-specific compression parameters and image-resolution metadata were not consistently retained in the archived records.

### 2.3. Reference Standard

The reference standard was established independently of the AI evaluations by two experienced orthodontists with 5 years of experience in orthodontics, who assessed all radiographs separately and were blinded to the outputs of the AI platforms. The basis of the reference classification differed according to the radiographic task. For lateral cephalometric radiographs, skeletal classification was derived from ANB measurements obtained using Dolphin Imaging, whereas hand–wrist and panoramic classifications were determined by visual application of the predefined categorical criteria described below. Interobserver agreement was assessed using Cohen’s kappa coefficient for lateral cephalometric classifications and linearly weighted Cohen’s kappa coefficient for the ordinal hand–wrist and panoramic classifications. Cases with disagreement were jointly reassessed, and the final classification reached by consensus was accepted as the reference standard.

### 2.4. Radiographic Classification Criteria

#### 2.4.1. Lateral Cephalometric Radiographs

Sagittal skeletal relationships were assessed using Steiner analysis performed with Dolphin Imaging Version 11.95.08.50 Premium (Dolphin Imaging & Management Solutions, Chatsworth, CA, USA) [4]. Final classification was based on the ANB angle: values of 0–4° were classified as skeletal Class I, values > 4° as skeletal Class II, and values < 0° as skeletal Class III. The AI platforms were not asked to identify cephalometric landmarks, calculate SNA, SNB, or ANB angles, or provide numerical cephalometric measurements. Accordingly, the lateral cephalometric task evaluated the ability of each model to infer the final ANB-derived skeletal classification directly from the radiographic image rather than its ability to perform a complete Steiner cephalometric analysis.

#### 2.4.2. Hand–Wrist Radiographs

Skeletal maturation was assessed using the Björk–Grave–Brown method [2]. For the categorical framework of this study, the original ossification indicators were grouped into three growth periods according to their temporal relationship with the pubertal growth spurt, rather than being evaluated as individual stages. The PP2=, MP3=, and H1 stages were classified as prepubertal; the S, H2, and MP3cap stages as pubertal; and the DP3u, PP3u, MP3u, and Ru stages as postpubertal.

#### 2.4.3. Panoramic Radiographs

The dental development stage was determined according to the eruption status of the permanent teeth and the presence of primary teeth [3]. Third molars were excluded from the assessment. Early mixed dentition was defined as the stage in which the permanent first molars and incisors had erupted, eruption of the permanent canines or premolars had not yet begun, and primary teeth were still present. Late mixed dentition was defined by the presence of at least one erupted permanent canine or premolar together with at least one retained primary tooth. Cases with no remaining primary teeth were classified as permanent dentition.

### 2.5. AI Platforms and Pilot Evaluation

Three general-purpose multimodal AI platforms were evaluated: ChatGPT–GPT-5.5 Instant (OpenAI, San Francisco, CA, USA), Gemini–Gemini 3.5 Flash (Google LLC, Mountain View, CA, USA), and Claude–Claude Sonnet 4.6 (Anthropic PBC, San Francisco, CA, USA). These platforms were selected because they represent independently developed general-purpose MLLMs with image-input capabilities that could be evaluated through comparable natural-language interactions using their standard web interfaces. All platforms were accessed from Türkiye through their standard web interfaces using individual user accounts, without API-based, developer, enterprise, or institutional access. No model settings or inference parameters were manually modified by the investigators. Evaluations were conducted sequentially according to radiographic task: lateral cephalometric radiographs were assessed between 29 May and 5 June 2026, panoramic radiographs between 6 and 13 June 2026, and hand–wrist radiographs between 14 and 21 June 2026. The model/version identifiers reported above correspond to those displayed in the respective web interfaces during these evaluation periods.

Before the main evaluation, a pilot assessment was conducted using 18 lateral cephalometric, 18 hand–wrist, and 18 panoramic radiographs that were not included in the study sample. For each radiograph type, the pilot set contained six images from each classification category (*n* = 6 per category). The pilot assessment confirmed the applicability of the standardized prompts. No modifications were made to the evaluation protocol after the pilot phase, and the pilot radiographs were excluded from the final analysis.

### 2.6. AI Evaluation Protocol

Each radiograph was uploaded separately to ChatGPT, Gemini, and Claude. To minimize potential contextual influence from previous assessments, every evaluation was conducted in a new chat session. No information regarding age, sex, clinical findings, diagnosis, treatment history, or other radiographs belonging to the same individual was provided to the AI platforms. Each session contained only one radiograph and its corresponding standardized prompt.

Identical standardized prompts in Turkish were used across all three AI platforms for each radiograph type. The platforms were instructed to return only one of the predefined classification categories. No example radiographs, reference classifications, additional explanations, or feedback were provided during the evaluations. Thus, the assessment followed a standardized zero-shot, single-image, single-pass protocol. Each radiograph was evaluated once by each AI platform. The standardized prompts are presented in Table 1.

**Figure 2:**
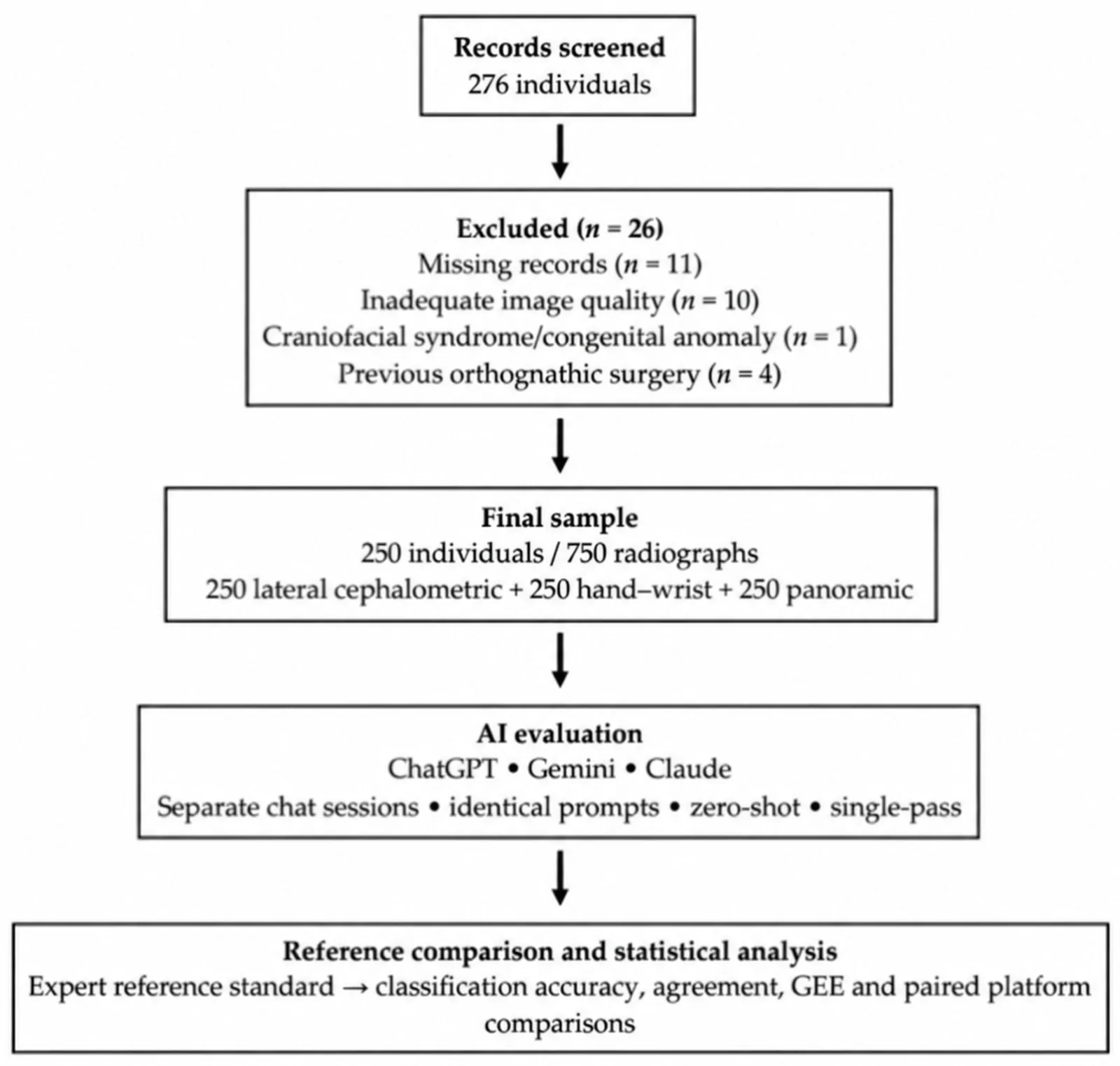
Flow diagram of participant selection, radiographic dataset formation, AI evaluation, and comparison with the reference standard.

The categorical outputs generated by each AI platform were recorded in an electronic database. Reference-standard classifications and AI-generated outputs were stored separately and matched only at the statistical analysis stage. All evaluations yielded a valid response corresponding to one of the predefined classification categories; no refusals, ambiguous or non-classifiable responses, or failed image uploads occurred. The overall study workflow, including participant selection, radiographic dataset formation, reference-standard assessment, AI evaluation, and subsequent comparison of classifications, is summarized in Figure 2.

### 2.7. Statistical Analysis

Sample size was calculated using G*Power 3.1. Because the same radiographs were evaluated by different AI platforms and the primary outcome was classification accuracy relative to the reference standard, the calculation was based on the McNemar test for paired proportions. Assuming a total discordant-pair proportion of 0.30, an odds ratio of 2.00, α = 0.05, and 80% power, the minimum required sample size was 240. Because directly applicable paired data were unavailable for these orthodontic radiographic tasks, the effect assumptions were specified a priori. This combination corresponds to discordant proportions of approximately 0.20 and 0.10, representing an absolute difference of approximately 10 percentage points in paired classification accuracy. A total of 250 individuals were included.

Statistical analyses were performed using IBM SPSS Statistics Version 26.0 (IBM Corp., Armonk, NY, USA). Continuous variables were summarized as mean ± standard deviation, and categorical variables as frequencies and percentages. For each radiograph type, AI classifications were compared with the reference standard using cross-tabulations and confusion matrices.

The primary outcome, classification accuracy, was defined as the proportion of cases in which the AI-generated classification matched the reference standard. Accuracy was reported with 95% confidence intervals (CIs) calculated using the Wilson method. Class-specific sensitivity, specificity, positive predictive value, negative predictive value, and F1 score were calculated for each AI platform and radiograph type. Because the reference-standard class distributions were unequal, balanced accuracy and macro-F1 were additionally reported alongside raw accuracy to provide a less distribution-dependent assessment of classification performance.

Generalized estimating equations (GEE) were used to assess the effects of AI platform, radiograph type, and their interaction on the probability of correct classification. Correct and incorrect classifications were coded as 1 and 0, respectively, and modeled using a binomial distribution with a logit link function. Repeated evaluations from the same individual were clustered at the individual level. The model included AI platform, radiograph type, and the platform × radiograph type interaction, with an exchangeable working correlation structure and robust standard errors. The a priori sample-size calculation was based on paired differences in classification accuracy between platforms and did not specifically address power for the platform × radiograph type interaction. Main effects and the interaction were evaluated using Wald chi-square tests and reported as Wald χ^2^ statistics, degrees of freedom, and *p* values. Because the platform × radiograph type interaction was significant, pairwise contrasts between AI platforms were additionally estimated within each radiograph type and reported as odds ratios (ORs) with 95% confidence intervals.

Agreement between each AI platform and the reference standard was assessed using Cohen’s kappa for lateral cephalometric radiographs and linearly weighted Cohen’s kappa for the ordinal hand–wrist and panoramic classifications. Linear weighting was selected to account for the ordered nature of these categories by assigning progressively greater weight to disagreements spanning more category levels, without assuming a quadratic increase in the importance of more distant disagreements. The same approach was used to assess interobserver agreement between the two orthodontists. Kappa coefficients for AI–reference standard agreement were reported with 95% CIs.

Within each radiograph type, paired classification accuracies of ChatGPT, Gemini, and Claude were compared using Cochran’s Q test. When the overall test was significant, pairwise comparisons were performed using McNemar tests with Bonferroni correction.

All statistical tests were two-sided, and *p* < 0.05 was considered statistically significant.

## 3. Results

A total of 250 individuals were included in the study, of whom 121 (48.4%) were male, and 129 (51.6%) were female. The mean age was 12.95 ± 2.57 years (range, 7.4–17.8 years).

Interobserver agreement between the two experienced orthodontists was high across all radiograph types. The kappa coefficients for lateral cephalometric, hand–wrist, and panoramic radiographs were κ = 0.93, κw = 0.91, and κw = 0.94, respectively.

For hand–wrist radiographs, the correct classification rates of ChatGPT for the prepubertal, pubertal, and postpubertal periods were 65.4%, 74.2%, and 97.3%, respectively. The corresponding rates were 50.0%, 64.5%, and 83.6% for Gemini and 64.1%, 54.8%, and 70.9% for Claude. The highest class-specific accuracy for ChatGPT was observed in postpubertal cases (97.3%). Gemini classified 34.6% of prepubertal cases as postpubertal, whereas Claude classified 45.2% of pubertal cases as postpubertal (Table 2).

The reference-standard distribution for panoramic radiographs comprised 27 early mixed, 38 late mixed, and 185 permanent dentition cases. For panoramic radiographs, the correct classification rates of ChatGPT for the early mixed, late mixed, and permanent dentition stages were 100.0%, 26.3%, and 90.8%, respectively. The corresponding rates were 100.0%, 92.1%, and 91.9% for Gemini and 77.8%, 60.5%, and 73.5% for Claude. ChatGPT classified 57.9% of late mixed dentition cases as early mixed dentition, whereas Claude classified 20.5% of permanent dentition cases as late mixed dentition (Table 3).

For lateral cephalometric radiographs, the correct classification rates of ChatGPT for Class I, Class II, and Class III cases were 34.5%, 84.7%, and 69.0%, respectively. The corresponding rates were 65.5%, 66.4%, and 58.6% for Gemini and 67.3%, 66.4%, and 58.6% for Claude. ChatGPT classified 43.6% of Class I cases as Class II, whereas both Gemini and Claude classified 36.2% of Class III cases as Class I (Table 4).

Based on the classification results presented in Table 2, Table 3 and Table 4, the overall classification performance of the AI platforms was evaluated (Table 5). For hand–wrist radiographs, the highest classification accuracy was achieved by ChatGPT (81.6%; 95% CI, 76.3–85.9), whereas Gemini showed the highest accuracy for panoramic radiographs (92.8%; 95% CI, 88.9–95.4). For lateral cephalometric radiographs, classification accuracies were 70.0%, 64.4%, and 64.8% for ChatGPT, Gemini, and Claude, respectively. The highest balanced accuracy and macro-F1 values were observed for ChatGPT on hand–wrist radiographs (79.0% and 0.788, respectively) and for Gemini on panoramic radiographs (94.7% and 0.900, respectively). For lateral cephalometric radiographs, balanced accuracy ranged from 62.7% to 64.1%, and macro-F1 ranged from 0.618 to 0.633. Class-specific sensitivity, specificity, positive predictive value, negative predictive value, and F1 scores are presented in Supplementary Table S1.

Generalized estimating equations (GEE) analysis identified significant main effects of AI platform (Wald χ^2^ = 30.95; df = 2; *p* < 0.001) and radiograph type (Wald χ^2^ = 42.55; df = 2; *p* < 0.001) on the probability of correct classification. Importantly, the platform × radiograph type interaction was also statistically significant (Wald χ^2^ = 42.52; df = 4; *p* < 0.001) (Table 6), indicating that the relative classification performance of the AI platforms varied across radiograph types. Task-specific pairwise GEE contrasts were therefore examined to quantify the magnitude and direction of these differences. For hand–wrist radiographs, ChatGPT had higher odds of correct classification than Gemini (OR = 2.05, 95% CI: 1.47–2.86) and Claude (OR = 2.41, 95% CI: 1.57–3.70), whereas the contrast between Gemini and Claude was smaller and its confidence interval included 1.00 (OR = 1.18, 95% CI: 0.84–1.65). For panoramic radiographs, ChatGPT had lower odds of correct classification than Gemini (OR = 0.35, 95% CI: 0.20–0.61) but higher odds than Claude (OR = 1.77, 95% CI: 1.22–2.57), while Gemini had substantially higher odds than Claude (OR = 5.01, 95% CI: 2.98–8.43). For lateral cephalometric radiographs, all pairwise 95% CIs included 1.00, indicating no clear between-platform differences.

To further examine the significant platform × radiograph type interaction identified in the GEE analysis, paired proportions of correct classifications were compared among the AI platforms within each radiograph type (Table 7). For hand–wrist radiographs, classification accuracy differed significantly among the platforms (*p* < 0.001), with ChatGPT showing significantly higher accuracy (81.6%) than Gemini (68.4%) and Claude (64.8%). A significant difference was also observed for panoramic radiographs (*p* < 0.001); Gemini showed significantly higher accuracy (92.8%) than ChatGPT (82.0%), and ChatGPT showed significantly higher accuracy than Claude (72.0%). In contrast, no significant difference was found among ChatGPT (70.0%), Gemini (64.4%), and Claude (64.8%) for lateral cephalometric radiographs (*p* = 0.336).

Agreement between the AI platforms and the reference standard varied across radiograph types (Table 8). For hand–wrist radiographs, the highest agreement was observed for ChatGPT (κw = 0.758; 95% CI, 0.692–0.824), whereas Gemini showed the highest agreement for panoramic radiographs (κw = 0.883; 95% CI, 0.830–0.937). For lateral cephalometric radiographs, agreement coefficients were comparable across ChatGPT, Gemini, and Claude, with κ values of 0.473, 0.449, and 0.452, respectively.

## 4. Discussion

In this study, the classification performance of ChatGPT, Gemini, and Claude was compared across lateral cephalometric, hand–wrist, and panoramic radiographs. The principal finding was a significant platform × radiograph type interaction, indicating that differences in classification performance among the AI platforms depended on the radiographic task being evaluated. Specifically, ChatGPT achieved the highest classification accuracy for hand–wrist radiographs, whereas Gemini showed the highest accuracy for panoramic radiographs; no significant difference among the platforms was observed for lateral cephalometric radiographs. These findings indicate that no single general-purpose multimodal large language model consistently outperformed the others across all orthodontic radiographic tasks and that classification performance was task-dependent. Accordingly, platform effects are more appropriately interpreted within individual radiographic tasks rather than as an overall ranking of the models. The significant platform, radiograph type, and interaction effects therefore led to rejection of the null hypothesis.

The differences observed across radiograph types may be related to variation in the anatomical structures and assessment criteria inherent to each radiograph type. Therefore, the findings should be considered separately for each radiographic task. Hand–wrist radiographs contain standardized anatomical indicators of skeletal maturation, including distinct and sequential morphological changes in the epiphyses, metaphyses, and sesamoid bones. The sequential appearance of the ossification indicators defined in the Björk–Grave–Brown approach may provide visually distinguishable patterns for AI-based image interpretation. Indeed, previous studies using task-specific AI models have reported high accuracy in skeletal maturation assessment from hand–wrist radiographs [15]. Yıldırım et al. compared different ChatGPT models and reported that GPT-4o demonstrated the highest level of agreement [16]. Akpınar et al. likewise reported successful skeletal maturation assessment from hand–wrist radiographs using ChatGPT-4.0, although its overall performance was lower than that of the other AI models evaluated [11]. In the present study, the significantly higher classification accuracy of ChatGPT compared with Gemini and Claude for hand–wrist radiographs may be related to differences in model versions, the grouping of maturation stages into three growth periods, and the evaluation protocol. This performance also varied across growth periods, with ChatGPT sensitivity increasing from 65.4% in prepubertal cases and 74.2% in pubertal cases to 97.3% in postpubertal cases. Accordingly, its overall accuracy of 81.6% should be interpreted in conjunction with the class-specific findings, particularly the markedly higher performance observed in the postpubertal category.

Nevertheless, previous studies have reported lower classification performance in some intermediate maturation stages around the pubertal growth spurt. Using a Björk-based assessment, Tentaş and Özden showed that performance was lower particularly for stages associated with the pubertal growth peak, such as S-H2 and MP3-Cap, compared with earlier and later maturation stages [17]. In the present study, although the highest correct classification rate was observed in the postpubertal period for all three AI platforms, the period with the lowest performance varied by platform: the lowest accuracy was observed in the prepubertal period for ChatGPT and Gemini, whereas it occurred in the pubertal period for Claude. This finding suggests that the difficulty of classifying maturation periods may vary according to the AI platform used. Because studies directly comparing ChatGPT, Gemini, and Claude using the same three-category growth-period classification are limited, the reasons underlying these interplatform differences cannot yet be fully explained based on the available literature.

In contrast to the findings for hand–wrist radiographs, Gemini demonstrated the highest classification performance for panoramic radiographs. Panoramic radiographs enable assessment of dentition stage by simultaneously displaying multiple anatomical indicators related to the presence of primary and permanent teeth, eruption status, and dental development. In our study, Gemini achieved not only the highest overall classification accuracy (92.8%) but also the highest balanced accuracy (94.7%) and macro-F1 (0.900) values for panoramic radiographs. These findings indicate more consistent performance across dentition categories. In comparison, although ChatGPT achieved an overall accuracy of 82.0%, its balanced accuracy was 72.4%, and macro-F1 was 0.644, reflecting substantial variation across dentition stages, with sensitivity and F1 for late mixed dentition of only 26.3% and 0.339, respectively, compared with 92.1% and 0.795 for Gemini. The difference between overall and class-balanced metrics is particularly relevant in the context of the unequal distribution of dentition categories in the study sample. Moreover, the low sensitivity observed for late mixed dentition may have clinical relevance because this transitional developmental stage is closely related to orthodontic treatment timing and planning. Studies evaluating general-purpose multimodal large language models on panoramic radiographs remain limited, with existing investigations focusing mainly on common dental findings, periodontal bone loss, and external apical root resorption [18,19]. Abuabara et al. reported numerically higher accuracy for ChatGPT than for Gemini in the assessment of external apical root resorption, although the difference between the two models was not statistically significant [12]. In contrast, in the present study, Gemini achieved significantly higher classification accuracy than both ChatGPT and Claude for determining the stage of dental development. The contrasting findings between studies may reflect differences in the tasks evaluated, classification criteria, and model versions.

Another notable finding was that ChatGPT frequently misclassified cases in the late mixed dentition stage as early mixed dentition. Differentiation between the early and late mixed dentition stages relies on the combined assessment of the presence of primary teeth and the eruption status of permanent teeth. One possible explanation is that individual variation in canine and premolar eruption may produce overlapping radiographic features between the early and late mixed dentition stages, thereby increasing classification difficulty. However, because the visual features prioritized by the models were not investigated, this explanation remains speculative.

In contrast to panoramic and hand–wrist radiographs, no significant difference in classification accuracy was found among the AI platforms for lateral cephalometric radiographs. This finding should be interpreted in the context of how the cephalometric task was defined. Although the reference classification was derived from ANB measurements, the AI platforms were asked to assign the final skeletal category directly from the radiographic image and were not required to identify landmarks or calculate SNA, SNB, or ANB angles. Thus, the reported accuracies reflect the models’ ability to infer an ANB-derived skeletal category rather than the accuracy of numerical cephalometric measurement or a complete Steiner analysis. Consequently, individual misclassifications cannot be attributed specifically to landmark-identification error, angular-measurement error, or incorrect application of the ANB classification thresholds. Determining sagittal skeletal class on lateral cephalometric radiographs requires assessment of the spatial relationships of the maxilla and mandible to the cranial base and to each other [20]. Even at the categorical level evaluated in the present study, this task therefore requires integration of multiple spatial features within the craniofacial complex. AI-based studies have suggested that skeletal classification may rely not only on specific jaw structures but also on broader visual information related to craniofacial morphology. Jeong et al. reported that skeletal class could be determined with high accuracy even on lateral cephalometric radiographs in which the mandible had been masked [21], suggesting that broader craniofacial information may contribute to automated skeletal classification. More broadly, AI-based cephalometric interpretation also depends on reliable recognition of craniofacial landmarks. Hendrickx et al. reported that mean errors in AI-assisted automated landmark detection were within clinically acceptable limits, while emphasizing that the available evidence should be interpreted cautiously because of a high risk of bias [22]. More recently, Nguyen et al. directly compared ChatGPT, Gemini, and Claude in a different cephalometric image-interpretation task involving lateral cephalometric superimpositions and reported that all three models performed worse than an orthodontic resident [14]. This finding suggests that general-purpose multimodal models may still have limitations in interpreting complex spatial relationships on cephalometric images.

Another noteworthy finding was the marked variation in ChatGPT performance across skeletal classes. Although its overall accuracy for lateral cephalometric radiographs was 70.0%, sensitivity was only 34.5% for Class I, compared with 84.7% for Class II and 69.0% for Class III. This class-dependent pattern was particularly evident in the frequent misclassification of Class I cases as Class II. Notably, Yıldırım et al. reported a tendency toward Class II classification across all GPT models when image-based prompts were used to evaluate lateral cephalometric radiographs [13], which is consistent with the Class II-directed error pattern observed in the present study. However, the present design did not evaluate which visual features contributed to the models’ classifications; therefore, the basis of this error pattern cannot be determined from these findings. In contrast, Kim et al., using a task-specific deep learning model, reported higher classification performance for Class I and Class II cases, with the lowest accuracy observed for Class III cases [23]. These differences between general-purpose multimodal models and task-specific AI systems suggest that model architecture, training data, and classification strategy may influence skeletal classification performance.

In addition to classification accuracy, assessment of agreement between the AI platforms and the reference standard provides a more comprehensive interpretation of model performance. Whereas classification accuracy reflects the proportion of correctly classified cases, the kappa coefficient evaluates observed agreement while accounting for agreement expected by chance. In the present study, agreement levels generally paralleled classification accuracy. For hand–wrist radiographs, ChatGPT achieved both the highest classification accuracy and the highest weighted kappa value, whereas the same pattern was observed for Gemini on panoramic radiographs. In contrast, for lateral cephalometric radiographs, kappa values were similar across ChatGPT, Gemini, and Claude, and no significant difference in classification accuracy was observed among the platforms. Taken together, these findings indicate that classification accuracy and agreement provide complementary information and should be interpreted alongside class-specific performance metrics when evaluating AI-based classification performance.

From a clinical perspective, the marked variability in performance across radiographic tasks is important. The relatively limited accuracy and agreement observed for lateral cephalometric classification do not support the use of these models as standalone tools for the orthodontic radiographic tasks evaluated in this study. Although higher performance was observed for selected tasks, particularly hand–wrist assessment with ChatGPT and panoramic assessment with Gemini, the clinical utility of these systems as decision-support tools was not directly evaluated. The class-specific variability observed despite relatively high aggregate performance in some tasks also warrants caution, as consistent-looking outputs may encourage over-reliance on model recommendations even when performance is weaker in particular diagnostic categories. Maintaining clinician oversight and responsibility for the final interpretation is therefore essential in any future clinical application [24,25]. Further prospective validation is therefore required before their integration into routine orthodontic diagnostic workflows. If used clinically, AI-generated assessments should remain subject to expert verification.

An important strength of this study is the direct comparison of three general-purpose multimodal large language models across three radiograph types routinely used in orthodontics, using the same sample and the same reference standard. The reference standard was established through independent assessments by two experienced orthodontists followed by consensus, and the high interobserver agreement provided a reliable reference for comparison. In addition, all radiographs were obtained at the same center using the same digital imaging system and standardized imaging protocols, thereby limiting variability related to image acquisition. The use of identical standardized prompts, performance of each assessment in a new chat session, and withholding of any additional clinical or demographic information from the models further enhanced comparability across platforms. Moreover, performance was evaluated not only in terms of classification accuracy, but also through agreement with the reference standard, balanced accuracy, macro-F1, and class-specific diagnostic metrics, while the interaction between AI platform and radiograph type was examined using a GEE approach. Together, these methodological features enabled a more comprehensive interpretation of model performance.

Nevertheless, this study has several limitations. First, it was conducted at a single center, and the sample represented a specific population; all radiographs were also acquired using the same imaging system. Therefore, external and multicenter validation across different populations, imaging systems, and clinical settings is needed to establish the generalizability of the findings. In addition, radiographs with inadequate image quality were excluded, which may limit generalizability to routine clinical settings where suboptimal images are encountered and may further affect model performance. Second, the evaluations were performed using the model versions available during the study period and a single standardized prompt in Turkish. Because general-purpose multimodal large language models are continuously updated and their performance may vary according to prompt structure and language, the present findings should be regarded as a snapshot of the specific deployed model versions evaluated under the access environment, web interface, prompt, language, and time period of this study rather than as fixed characteristics of the respective platforms. Differences in these conditions or subsequent model updates may therefore affect external reproducibility. Furthermore, the image-only evaluation protocol, although useful for standardizing comparisons across platforms, does not reproduce the multimodal clinical context in which orthodontic diagnosis is typically performed. In addition, the radiographs were exported from DICOM to JPEG for AI evaluation, and export-specific compression and resolution parameters were not available; therefore, the potential influence of this conversion on image detail could not be assessed. Although no investigator-applied resizing, cropping, or image enhancement was performed, the commercial web interfaces may have applied platform-specific image preprocessing, such as rescaling or compression, during upload. These processes were not directly controllable or verifiable and may have influenced image presentation to the models.

Third, each radiograph was evaluated only once by each AI platform; therefore, the stability of classifications across repeated independent sessions and intra-model reproducibility could not be assessed. Given the potentially variable nature of generative model outputs, repeated evaluations of the same image may not necessarily yield identical classifications. Accordingly, the reported performance estimates should be interpreted as reflecting the models’ outputs under the specific single-pass evaluation conditions of this study, and between-platform differences may vary across repeated independent runs. This should be considered when interpreting model reliability and comparative performance. In addition, the classification tasks were reduced to predefined categorical outcomes, and grouping skeletal maturation and dental development into broader clinically meaningful categories precluded assessment of the models’ performance across more detailed individual stages or more nuanced clinical interpretations. The unequal distribution of reference categories, particularly for panoramic radiographs, may also have influenced aggregate accuracy estimates despite the additional reporting of balanced accuracy and macro-F1. More broadly, these constrained image-based classification tasks do not represent comprehensive orthodontic diagnosis, which ordinarily integrates radiographic findings with clinical and other diagnostic information. Finally, only general-purpose multimodal large language models were investigated; therefore, the findings do not reflect the performance of task-specific AI systems developed specifically for orthodontic image analysis.

Future studies should externally validate these models in larger, multicenter samples acquired using different imaging systems and investigate the effects of model version, prompt structure, language, and repeated independent evaluations of the same images on classification performance. Direct comparisons between general-purpose MLLMs and task-specific orthodontic AI systems using the same datasets would also help clarify their relative strengths and potential clinical applications. For lateral cephalometric assessment specifically, future studies could extend the present categorical approach by evaluating whether MLLMs can identify relevant landmarks and generate numerical SNA, SNB, and ANB measurements, allowing direct comparison with expert- or software-derived cephalometric measurements. Prospective studies should also determine whether AI assistance improves clinician performance compared with unaided assessment in real clinical workflows.

## 5. Conclusions

The classification performance of general-purpose multimodal large language models varied according to both the AI platform and the predefined orthodontic radiographic task. ChatGPT achieved the highest classification accuracy for hand–wrist radiographs, whereas Gemini showed the highest accuracy for panoramic radiographs; no significant difference among the platforms was observed for lateral cephalometric radiographs. The significant platform × radiograph type interaction confirmed that relative model performance was task-dependent and that no single platform consistently outperformed the others.

For the radiographic classification tasks evaluated in this study, the findings do not support the use of general-purpose multimodal large language models as standalone tools. Although relatively high performance was observed for selected tasks, their clinical value in orthodontic decision-making remains to be established. Prospective studies comparing clinician performance with and without AI assistance, together with external validation, are required before these systems can be considered for routine orthodontic practice.

## Figures and Tables

**Table 1:** Standardized prompts used for AI-based radiographic classification.

Radiograph Type	Standardized Prompt
Lateral cephalometric radiograph	Evaluate this lateral cephalometric radiograph from an orthodontic perspective. Determine the individual’s sagittal skeletal relationship according to the Steiner analysis. Your response must consist of only one of the following options: Class I, Class II, or Class III. Do not provide any explanation, justification, measurement, or additional information.
Hand–wrist radiograph	Evaluate this hand–wrist radiograph. Determine the individual’s growth period according to the Björk–Grave–Brown method. Your response must consist of only one of the following options: Prepubertal, Pubertal, or Postpubertal. Do not provide any explanation, justification, or additional information.
Panoramic radiograph	Evaluate this panoramic radiograph. Determine the stage of dental development. Your response must consist of only one of the following options: Early mixed dentition, Late mixed dentition, or Permanent dentition. Do not provide any explanation, justification, or additional information.

The original prompts were administered to all AI platforms in Turkish; English translations are presented here for publication.

**Table 2:** Distribution of growth-period classifications by AI platforms on hand–wrist radiographs according to the reference standard.

AI Platform	AI Classification	Prepubertal *n* (%)	Pubertal *n* (%)	Postpubertal *n* (%)
ChatGPT	Prepubertal	51 (65.4)	13 (21.0)	3 (2.7)
	Pubertal	21 (26.9)	46 (74.2)	0 (0.0)
	Postpubertal	6 (7.7)	3 (4.8)	107 (97.3)
Gemini	Prepubertal	39 (50.0)	0 (0.0)	0 (0.0)
	Pubertal	12 (15.4)	40 (64.5)	18 (16.4)
	Postpubertal	27 (34.6)	22 (35.5)	92 (83.6)
Claude	Prepubertal	50 (64.1)	0 (0.0)	3 (2.7)
	Pubertal	9 (11.5)	34 (54.8)	29 (26.4)
	Postpubertal	19 (24.4)	28 (45.2)	78 (70.9)

Data are presented as *n* (%). Percentages represent the distribution within each reference-standard category. Diagonal cells indicate correct classifications, whereas off-diagonal cells indicate misclassifications.

**Table 3:** Distribution of dental development stage classifications by AI platforms on panoramic radiographs according to the reference standard.

AI Platform	AI Classification	Early Mixed *n* (%)	Late Mixed *n* (%)	Permanent *n* (%)
ChatGPT	Early mixed	27 (100.0)	22 (57.9)	6 (3.2)
	Late mixed	0 (0.0)	10 (26.3)	11 (5.9)
	Permanent	0 (0.0)	6 (15.8)	168 (90.8)
Gemini	Early mixed	27 (100.0)	3 (7.9)	0 (0.0)
	Late mixed	0 (0.0)	35 (92.1)	15 (8.1)
	Permanent	0 (0.0)	0 (0.0)	170 (91.9)
Claude	Early mixed	21 (77.8)	6 (15.8)	11 (5.9)
	Late mixed	6 (22.2)	23 (60.5)	38 (20.5)
	Permanent	0 (0.0)	9 (23.7)	136 (73.5)

Data are presented as *n* (%). Percentages represent the distribution within each reference-standard category. Diagonal cells indicate correct classifications, whereas off-diagonal cells indicate misclassifications.

**Table 4:** Distribution of skeletal classifications by AI platforms on lateral cephalometric radiographs according to the reference standard.

AI Platform	AI Classification	Class I *n* (%)	Class II *n* (%)	Class III *n* (%)
ChatGPT	Class I	19 (34.5)	9 (6.6)	0 (0.0)
	Class II	24 (43.6)	116 (84.7)	18 (31.0)
	Class III	12 (21.8)	12 (8.8)	40 (69.0)
Gemini	Class I	36 (65.5)	40 (29.2)	21 (36.2)
	Class II	6 (10.9)	91 (66.4)	3 (5.2)
	Class III	13 (23.6)	6 (4.4)	34 (58.6)
Claude	Class I	37 (67.3)	29 (21.2)	21 (36.2)
	Class II	9 (16.4)	91 (66.4)	3 (5.2)
	Class III	9 (16.4)	17 (12.4)	34 (58.6)

Data are presented as *n* (%). Percentages represent the distribution within each reference-standard category. Diagonal cells indicate correct classifications, whereas off-diagonal cells indicate misclassifications.

**Table 5:** Overall classification performance of the AI platforms according to radiograph type.

Radiograph Type	AI Platform	Correct Classification *n* (%)	95% CI	Balanced Accuracy (%)	Macro-F1
Hand–wrist	ChatGPT	204 (81.6)	76.3–85.9	79.0	0.788
	Gemini	171 (68.4)	62.4–73.8	66.1	0.669
	Claude	162 (64.8)	58.7–70.5	63.3	0.645
Panoramic	ChatGPT	205 (82.0)	76.8–86.3	72.4	0.644
	Gemini	232 (92.8)	88.9–95.4	94.7	0.900
	Claude	180 (72.0)	66.1–77.2	70.6	0.636
Lateral cephalometric	ChatGPT	175 (70.0)	64.1–75.3	62.7	0.633
	Gemini	161 (64.4)	58.3–70.1	63.5	0.618
	Claude	162 (64.8)	58.7–70.5	64.1	0.619

CI: confidence interval. The 95% CIs for classification accuracy were calculated using the Wilson method. Balanced accuracy represents the mean sensitivity across classes, whereas macro-F1 represents the unweighted mean of class-specific F1 scores.

**Table 6:** GEE analysis of the effects of AI platform and radiograph type on the probability of correct classification, with task-specific pairwise contrasts.

Effect/Contrast	Wald χ^2^	df	*p*	OR	95% CI
Overall GEE effects					
AI platform	30.95	2	<0.001	—	—
Radiograph type	42.55	2	<0.001	—	—
Platform × radiograph type	42.52	4	<0.001	—	—
Task-specific pairwise GEE contrasts					
Hand–wrist					
ChatGPT vs. Gemini	—	—	—	2.05	1.47–2.86
ChatGPT vs. Claude	—	—	—	2.41	1.57–3.70
Gemini vs. Claude	—	—	—	1.18	0.84–1.65
Panoramic					
ChatGPT vs. Gemini	—	—	—	0.35	0.20–0.61
ChatGPT vs. Claude	—	—	—	1.77	1.22–2.57
Gemini vs. Claude	—	—	—	5.01	2.98–8.43
Lateral cephalometric					
ChatGPT vs. Gemini	—	—	—	1.29	0.85–1.97
ChatGPT vs. Claude	—	—	—	1.27	0.87–1.84
Gemini vs. Claude	—	—	—	0.98	0.71–1.35

GEE: generalized estimating equations; OR: odds ratio; CI: confidence interval. The model used a binomial distribution with a logit link function. Repeated evaluations from the same individual were clustered at the individual level using an exchangeable working correlation structure and robust standard errors. Task-specific pairwise contrasts were estimated because the platform × radiograph type interaction was significant. An OR > 1 indicates higher odds of correct classification for the first-listed platform.

**Table 7:** Comparison of paired correct-classification rates among AI platforms according to radiograph type.

Radiograph Type	ChatGPT *n* (%)	Gemini *n* (%)	Claude *n* (%)	Cochran’s Q *p*	Bonferroni-Adjusted McNemar Comparisons
Hand–wrist	204 (81.6)	171 (68.4)	162 (64.8)	<0.001	ChatGPT > Gemini, Claude
Panoramic	205 (82.0)	232 (92.8)	180 (72.0)	<0.001	Gemini > ChatGPT > Claude
Lateral cephalometric	175 (70.0)	161 (64.4)	162 (64.8)	0.336	—

Correct classification indicates cases in which the AI-generated classification matched the reference standard. The reported *p* values are from Cochran’s Q test. For radiograph types with a significant overall Cochran’s Q test, pairwise comparisons were performed using McNemar tests with Bonferroni adjustment. The “>” symbol indicates a statistically significantly higher correct-classification rate.

**Table 8:** Agreement between AI platform classifications and the reference standard.

Radiograph Type	ChatGPT κ/κw (95% CI)	Gemini κ/κw (95% CI)	Claude κ/κw (95% CI)
Hand–wrist	0.758 (0.692–0.824)	0.517 (0.426–0.609)	0.507 (0.414–0.600)
Panoramic	0.703 (0.626–0.780)	0.883 (0.830–0.937)	0.530 (0.432–0.627)
Lateral cephalometric	0.473 (0.379–0.568)	0.449 (0.363–0.535)	0.452 (0.365–0.539)

κ: Cohen’s kappa coefficient; κw: linearly weighted Cohen’s kappa coefficient; CI: confidence interval. Linearly weighted Cohen’s kappa was used for the ordinal hand–wrist and panoramic classifications, whereas unweighted Cohen’s kappa was used for lateral cephalometric classifications.

## Data Availability

The data supporting the findings of this study are available from the corresponding author upon reasonable request. The data are not publicly available due to privacy and ethical restrictions related to the use of archived patient records.

## Supplementary Materials

The following supporting information can be downloaded at: https://www.mdpi.com/article/10.3390/medicina62091714/s1, Table S1: Class-specific diagnostic performance metrics of the artificial intelligence platforms according to radiograph type.

## Abbreviations

AI	Artificial intelligence
MLLM	Multimodal large language model
GEE	Generalized estimating equations
CI	Confidence interval
PPV	Positive predictive value
NPV	Negative predictive value
ANB	A–Nasion–B angle
