# Supplementary material for: Classification Performance of General-Purpose Multimodal Large Language Models Across Orthodontic Radiographic Tasks: A Comparative Study of ChatGPT, Gemini, and Claude
Source: Medicina (Kaunas). 2026 Sep 6;62(9):1714. doi: 10.3390/medicina62091714 (PMC13609597; doi:10.3390/medicina62091714)
Supplement: Supplementary file 1 [file medicina-62-01714-s001.zip › medicina-4532237-supplementary.pdf]

Supplementary Table S1. Class-specific diagnostic performance metrics of the artificial intelligence platforms according to radiograph type

Radiograph type	Platform	Class	Sensitivity (%)	Specificity (%)	PPV (%)	NPV (%)	F1
Hand–wrist	ChatGPT	Prepubertal	65.4	90.7	76.1	85.2	0.703
		Pubertal	74.2	88.8	68.7	91.3	0.713
		Postpubertal	97.3	93.6	92.2	97.8	0.947
	Gemini	Prepubertal	50.0	100.0	100.0	81.5	0.667
		Pubertal	64.5	84.0	57.1	87.8	0.606
		Postpubertal	83.6	65.0	65.2	83.5	0.733
	Claude	Prepubertal	64.1	98.3	94.3	85.8	0.763
		Pubertal	54.8	79.8	47.2	84.3	0.507
		Postpubertal	70.9	66.4	62.4	74.4	0.664
Panoramic	ChatGPT	Early mixed	100.0	87.4	49.1	100.0	0.659
		Late mixed	26.3	94.8	47.6	87.8	0.339
		Permanent	90.8	90.8	96.6	77.6	0.936
	Gemini	Early mixed	100.0	98.7	90.0	100.0	0.947
		Late mixed	92.1	92.9	70.0	98.5	0.795
		Permanent	91.9	100.0	100.0	81.2	0.958
	Claude	Early mixed	77.8	92.4	55.3	97.2	0.646
		Late mixed	60.5	79.2	34.3	91.8	0.438
		Permanent	73.5	86.2	93.8	53.3	0.824
Lateral cephalometric	ChatGPT	Class I	34.5	95.4	67.9	83.8	0.458
		Class II	84.7	62.8	73.4	77.2	0.786
		Class III	69.0	87.5	62.5	90.3	0.656
	Gemini	Class I	65.5	68.7	37.1	87.6	0.474
		Class II	66.4	92.0	91.0	69.3	0.768
		Class III	58.6	90.1	64.2	87.8	0.613
	Claude	Class I	67.3	74.4	42.5	89.0	0.521
		Class II	66.4	89.4	88.3	68.7	0.758
		Class III	58.6	86.5	56.7	87.4	0.576

PPV: positive predictive value; NPV: negative predictive value.
